# Biofilm-Forming Capacity and Drug Resistance of Different *Gardnerella* Subgroups Associated with Bacterial Vaginosis

**DOI:** 10.3390/microorganisms11092186

**Published:** 2023-08-30

**Authors:** Hanyu Qin, Yun Liu, Zhengyuan Zhai, Bingbing Xiao

**Affiliations:** 1 Department of Obstetrics and Gynecology, Peking University First Hospital, Beijing 100034, China; 2 College of Food Science and Nutritional Engineering, China Agricultural University, Beijing 100083, Chinazhaizy@cau.edu.cn (Z.Z.)

**Keywords:** *Gardnerella*, bacterial vaginosis, cpn60 sequencing, biofilm, resistance, virulence factor

## Abstract

Bacterial vaginosis (BV) is the most common infection of the lower reproductive tract among women of reproductive age. Recurrent infections and antibiotic resistance associated with biofilms remain significant challenges for BV treatment. *Gardnerella* species are commonly found in women with and without BV, indicating that genetic differences among *Gardnerella* isolates may distinguish pathogenic from commensal subgroups. This study isolated 11 *Gardnerella* strains from vaginal samples obtained from women with BV before or after treatment. The biofilm formation ability of each strain was examined by crystal violet staining. Eight strains were selected using phylogenetic analysis of the cpn60 sequences and classified as subgroups A (6/8), B (1/8), and D (1/8). The biofilm formation ability and antibiotic resistance profile of these strains was compared among the subgroups. Subgroup D had the strongest biofilm formation ability. Six of the planktonic strains exhibited resistance to the first-line BV drug, metronidazole, and one to clindamycin. Moreover, biofilm formation in vitro increased strain resistance to clindamycin. Two strains with strong biofilm ability, S20 and S23, and two with weak biofilm ability, S24 and S25, were selected for comparative genomic analysis. S20 and S23 were found to contain four key genes associated with biofilm formation and more genes involved in carbohydrate synthesis and metabolism than S24 and S25. Identifying differences in the expression of virulence factors between *Gardnerella* subgroups could inform the development of novel treatments for BV.

## 1. Introduction

Bacterial vaginosis (BV) is a common lower reproductive tract infection in women of reproductive age, affecting up to 58% of women worldwide, with a disproportionately high impact on those living in sub-Saharan Africa [1]. BV is characterized by a sharp decline in *Lactobacillus* counts, altered polymicrobial interactions, and an increase in the concentration of facultative or obligate anaerobic microbes [2,3,4]. Current Centers for Disease Control and Prevention (CDC) guidelines recommend metronidazole as a first-line treatment [5]. The clinical cure rates of different clinical treatments and routes of administration vary from 46.75% to 96.20% [6]. However, BV recurrence rates within 12 months of treatment remain above 70% [7]. The hallmark of BV is the presence of a highly structured polymicrobial biofilm on the vaginal epithelium that is primarily composed of *Gardnerella* spp. and other anaerobic species [8]. The overgrowth of vaginal biofilms seeded by initial *Gardnerella* spp. colonization results in recurrent symptomatic BV that has limited responsiveness to classical antibiotics [9].

The role of *Gardnerella* spp. in reproductive health remains incompletely understood. Some studies indicate that every *Gardnerella* spp. has the potential to cause BV, while others suggest that certain *Gardnerella* strains are genetically driven toward a more pathogenic phenotype [10]. While both BV- and non-BV-associated *Gardnerella* isolates contain virulence factors, BV-associated *Gardnerella* isolates appear to have a higher capacity for adherence and increased cytotoxicity [11,12]. Advanced molecular approaches have identified several novel *Gardnerella* species with distinct virulence potential [13]. *Gardnerella vaginalis* was the only recognized species for four decades; however, an amended description of *Gardnerella* spp. and descriptions of three new species, *G. leopoldii*, *G. piotii*, and *G. swidsinskii*, have been proposed [14]. Using PCR amplification and sequencing of the ‘universal target’ region of the 60-kDa chaperonin (cpn60) gene, *Gardnerella* spp. has been divided into four subgroups (A, B, C, and D) [15,16]. Phylogenetic and in vitro investigations suggest differences in their ecological and virulence properties [17].

*Gardnerella* spp. possess several virulence factors that can contribute to their pathogenic phenotype, including a hemolysin, mucus-degrading sialidases, resistance factors, and those involved in biofilm formation [18,19]. These factors have been extensively studied, though an understanding of the virulence potential of each subgroup remains insufficient [12,20,21,22]. The current study used the cpn60 sequence to compare biofilm formation, resistance, and the expression of biofilm-forming genes among various cpn60 subgroups. Functional analysis of the virulence potential of *Gardnerella* spp. using in vitro assays suggested that the most virulent *Gardnerella* spp. are responsible for poor BV treatment outcomes [11,21].

This study examined virulence factors from 11 *Gardnerella* strains isolated from vaginal samples. The biofilm-forming capacity and resistance of *Gardnerella* isolates were compared among cpn60 subgroups using an in vitro model. While the number of experimental strains was limited, the findings lay a foundation for future studies of BV pathogenesis and *Gardnerella* spp. virulence factors. The research of more efficacious BV treatments is clearly needed, particularly those that disrupt biofilms and target the most pathogenic bacteria.

## 2. Materials and Methods

### 2.1. Collection of Vaginal Specimens and Ethical Approval

A total of 11 women with BV who were patients in the Department of Gynecology at Peking University First Hospital were enrolled in the study and signed informed consent. The women included eight patients with BV (Nugent score of 7–10), two healthy women (Nugent score of 0–3), and one patient with intermediate BV (Nugent score of 4–6). The Ethics Committee of Peking University First Hospital in Beijing approved this study (V2.0/2020.04.20), which was performed in accordance with the Declaration of Helsinki. Eligible women with BV met at least three of four Amsel criteria and had a Nugent score of 7–10 [23,24].

All subjects were asked to provide two vaginal samples, one pre-treatment sample for strain isolation, and another to evaluate the efficacy of metronidazole treatment. The vaginal swabs were obtained from a standard anatomical site (lateral vaginal wall). One vaginal swab was sent to the microbiology laboratory for Gram staining while the second was placed in 1 mL of PBS and stored at −80 °C for genomic DNA extraction. The BV patients were treated with a standard 5-day metronidazole course (0.75% metronidazole gel vaginal administration from NiMeiXin, Tongfang Pharmaceutical Group Co., Ltd., Beijing, China, 5 g once daily) [5].

### 2.2. Culture Conditions and Strain Isolation

The vaginal swabs were inoculated onto a goat blood agar medium plate using the three-line method and cultured at 37 °C under anaerobic conditions (5% CO_2_, 95% nitrogen) for 48–72 h. Suspected colonies were purified onto blood agar medium for 48–72 h. After several purification cultures, 11 *Gardnerella* strains were completely isolated from BV patient vaginal samples.

Sheep blood agar plate: double anti-chocolate agar medium (22 g) was dissolved in 500 mL distilled water and mixed in a 500 mL triangular bottle. Under high pressure, the solution was cooled to 50 °C in a water bath, and 25 mL of sheep blood was added, mixed, and poured into a disposable Petri dish.

Supplemented brain–heart infusion broth (sBHI) medium: 18.5 g BHI basal medium, 1.5 g soluble starch, and 1.5 g glucose were added to 500 mL distilled water and autoclaved at 121 °C.

### 2.3. Strain Identification

After several purification cultures, single colonies were selected from the blood agar plates and added to 20 μL sterile ddH_2_O. The colonies were mixed with a pipetting gun, treated in a microwave at high temperature for 3 min, and incubated in an ice bath for 2 min. The resulting mixed system was a suspension of lysed bacteria, which was used as a colony PCR template. To identify the isolates, the 16S rDNA sequence primers, 16S rDNA-F (AGAGTTTGATCCTGGCTCAG) and 16S rDNA-R (TACGGTTACCTTGTTACGACTT), were used. The isolates were confirmed by comparing the 16S rDNA sequences with the GenBank library using the BLAST program (https://blast.ncbi.nlm.nih.gov (accessed on 10 June 2021)). The PCR amplification procedure included 30 cycles of pre-denaturation at 98 °C for 3 min, denaturation at 98 °C for 30 s, annealing at 52.8 for 30 s, and extension at 72 °C for 50 s, followed by a final extension at 72 °C for 10 min. PCR amplification products were detected using electrophoresis on a 1% agarose gel. Qualified products were sequenced by Beijing Qingke Xinye Biotechnology Co., Ltd. (Beijing, China), and the sequencing results were submitted to the online BLAST database for comparison.

### 2.4. In Vitro Biofilm Formation and Quantification at Different Stages

The clinically isolated *Gardnerella* strains were adjusted to a turbidity of 0.5 using a turbidimeter and inoculated into a 96-well plate (200 μL/well). The solution (2 μL) was inoculated into 198 μL of sBHI broth and incubated anaerobically at 37 °C in 96-well plates for 48 h. The bacterial solution was discarded, and the planktonic bacteria were removed by slowly rinsing three times with phosphate-buffered saline (PBS). The wells were air-dried for 60 min, and the biofilms were stained with 200 μL 0.4% (*w*/*v*) crystal violet for 30 min. The stains were removed by light washing with 200 μL PBS and air drying for 5 min. Crystal violet was dissolved in 200 μL of a 33% (*v*/*v*) acetic acid solution. The absorbance of crystal violet at 595 nm was measured using a microplate reader (Infinite M200 PRO, TECAN). Three biofilm formation experiments were performed for each strain and the average value of the three experiments was recorded. The liquid sBHI medium without bacteria was used as the control group. The OD cut-off value (ODc) was defined as three standard deviations (SD) above the mean OD of the negative control: ODc = average OD of negative control + (3 × SD of negative control), OD ≤ ODc = non biofilm producer; ODc < OD ≤ 2 × ODc = weak biofilm producer; 2 × ODc < OD ≤ 4 × ODc = moderate biofilm producer; 4 × ODc < OD = strong biofilm producer [25].

The activated strain was inoculated into sBHI broth at a 1% concentration. The OD_600_ value was measured every 2 h until it was stable. This method was used to describe the 24 h growth curve of *Gardnerella* spp. in liquid media. The concentration of the bacterial solution was adjusted to a turbidity of 0.5, and 2 μL of the solution was inoculated into 198 μL of sBHI broth. The solution was anaerobically cultured at 37 °C in a 96-well plate for 24 h, 48 h, 72 h, and 96 h, and the amount of biofilm was determined by crystal violet staining.

### 2.5. cpn60 Sequencing of Gardnerella Strains and Detection of Virulence Factors

Three samples with unqualified DNA samples were excluded, allowing only eight strains to be sequenced for cpn60. The DNA of the strains was used as the PCR template, and the primers, cpn60 F (5′-ATGGCAAAGATTATTGCCTATGAAG-3′) and cpn60 R (5′-GCATTCTGTAGAGCAGAACGAGT-3′), were used to amplify and type the cpn60 isolates. The PCR amplification procedure and electrophoresis used the same methods described above. The results were observed using a gel imager, and the amplified samples were sent for sequencing and the detection of virulence factors (Tsingke Biotechnology Co., Ltd., Beijing, China). Sequences of the virulence factors are shown in Supplementary Table S1.

There has been little success in connecting the genotypic with phenotypic characteristics, or in identifying any genotype or phenotype with clinical significance. HMPREF0424_0103 is the vaginolysin (VLY) gene, which is cytotoxic, destroys the integrity of vaginal epithelial cells, and is considered to be associated with the pathogenicity of *Gardnerella* spp. [21]. HMPREF0424_1109 is the sialidase gene, which hydrolyzes sialic acid in the mucosa and promotes the destruction of the protective mucous layer of vaginal epithelium. This process may also promote the adhesion of BV-associated species on the vaginal epithelium, leading to the biofilm formation [26]. HMPREF0424_0125 encodes the assembly of TadE/G-like family proteins and is considered to play an important role in its adhesion to vaginal epithelial cells [27]. HMPREF0424_0821 encodes a glycosyltransferase that is required for the biosynthesis of extracellular polysaccharides and is closely related to the formation of biofilms [28].

### 2.6. Minimal Inhibitory Concentration Assays

The minimum inhibitory concentration (MIC) for metronidazole and clindamycin was defined as the lowest antibiotic concentration that produced a marked reduction or inhibition in growth. Using the Clinical Laboratory Standards Institute (CLSI) guidelines, the sensitivity and resistance of eight *Gardnerella* strains to metronidazole and clindamycin were determined in the planktonic cells and biofilms using the broth microdilution method [29].

The final concentrations of metronidazole and clindamycin for planktonic bacteria were 0.125–256 μg/mL and 0.0625–128 μg/mL, respectively. The *Gardnerella* strains were anaerobically incubated on blood plates for 48–72 h. Single colonies of the *Gardnerella* strains were moved from the blood plates to sBHI and the concentration of the bacterial suspension was adjusted to 10^8^ CFU/mL (OD_595_ = 0.5) (Thermo Fisher Scientific, Waltham, MA, USA). A 100 μL solution composed of 2 × 10^6^ CFU/mL was mixed with different concentrations of the drug and bacterial suspension at a 1:1 ratio, inoculated on a 96-well plate, and incubated for 48–72 h. The preparation and inoculation process was completed within 15 min. According to the CLSI, the two antibiotics have the following critical values: metronidazole (sensitivity: MIC ≤ 8 μg/mL; intermediate: MIC = 16 μg/mL; drug-resistant: MIC ≥ 32 μg/mL) and clindamycin (sensitivity: MIC ≤ 2 μg/mL; intermediate: MIC = 4 μg/mL; resistant: MIC ≥ 8 μg/mL). The sBHI sample with bacterial growth and no antibiotic was used as the control group.

### 2.7. Statistical Analysis

SPSS19.0 software was used for statistical analyses. The Mann–Whitney test was used to analyze the differences in the biofilm formation ability of the cpn60 subgroups. The MIC of *Gardnerella* strains to metronidazole and clindamycin before and after biofilm formation was assessed using a *T*-test. *p* ≤ 0.05 indicated statistical significance.

## 3. Results

### 3.1. Clinical Characteristics of the Gardnerella Strains

The demographic and clinical characteristics of the 11 *Gardnerella* strains were generally well-balanced at baseline (Table 1). The strains were isolated from BV, BV intermediate, and healthy vaginal samples, even after metronidazole treatment, indicating that metronidazole is unable to completely eliminate *Gardnerella* spp. in the vagina. While evidence indicates that BV can be cured, clinical symptoms can persist for some patients.

### 3.2. cpn60 Typing of Gardnerella Strains and Biofilm Formation

**Figure 1:**
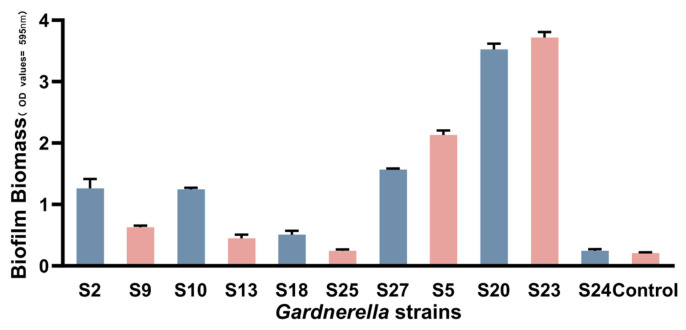
Biofilm formation ability of 11 *Gardnerella* strains. Biomass quantification of *Gardnerella* strains using the crystal violet method.

The phylogenetic tree divided *Gardnerella* into three subgroup, A, B, and D. Six (75%) strains belonged to subgroup A, one (12.5%) belonged to subgroup B, one (12.5%) belonged to subgroup D, and none belonged to subgroup C. The biofilm formation ability of the strains was compared (Figure 1 and Table 2) and ranked from strong to weak as follows: S23, S20, S5, S27, S2, S10, S9, S18, S13, S24, and S25 (*p* < 0.005). The subgroups were ranked from strong to weak by their ability to form biofilms. Subgroup D had the strongest ability to form biofilms, followed by subgroups B and A (Table 2).

### 3.3. Growth Curves of the Gardnerella Strains

**Figure 2:**
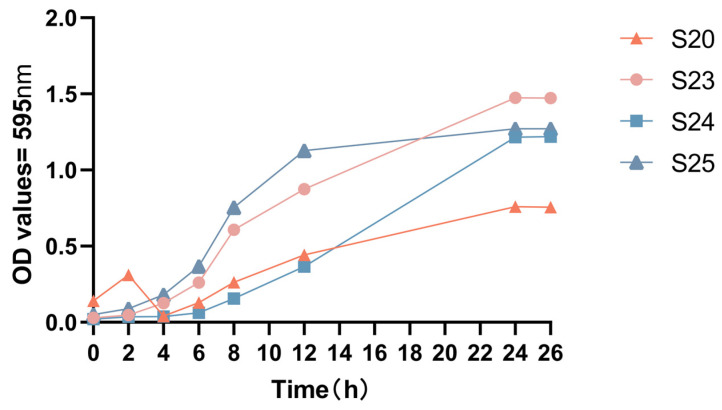
Growth curve of the four *Gardnerella* strains. The strains are ranked from fast to slow growth: S20, S23, S24, and S25.

The strains with the strongest biofilm-forming ability, S23 and S20, and those with the weakest ability, S24 and S25, were selected to determine the 24-h growth curves (Figure 2). *Gardnerella* strains reached a stable period after 24 h. The OD value of S23 was highest at 24 h and reached 1.5. Meanwhile, the OD values of S24 and S25 were 1.25 and 1.3, respectively (Figure 1). S20 had the lowest growth rate but strong biofilm-forming ability (Figure 1), suggesting that the ability to form biofilms is not related to the growth activity of *Gardnerella* isolates.

### 3.4. Quantification of Gardnerella Biofilm Formation at Different Times

**Figure 3:**
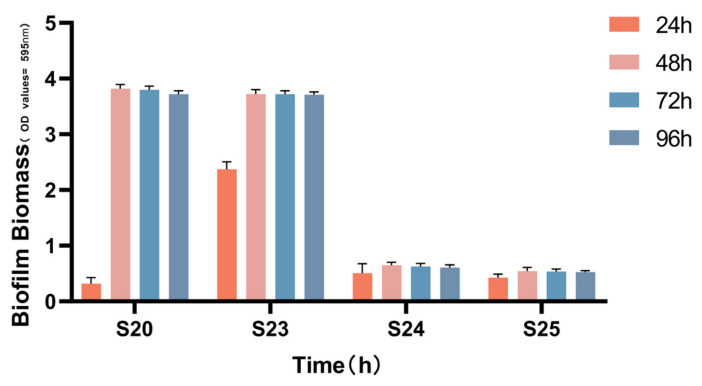
The amount of biofilm produced by the four *Gardnerella* strains over time. The amount of biofilm produced by the four *Gardnerella* strains reached a maximum at 48 h and 96 h.

The biofilm formation of S20, S23, S24, and S25 was measured at 24 h, 48 h, 72 h and 96 h (Figure 3). S23 had the fastest ability to form biofilms. The amount of biofilm created by each strain reached its highest value by 48 h and did not increase at 72 h and 96 h. These findings indicate that *Gardnerella* spp. biofilms reach a steady state in vitro at 48 h and do not spontaneously dissolve during continuous cultivation.

### 3.5. The MIC of Gardnerella *spp.* Planktonic Cells and Biofilms in Response to Metronidazole and Clindamycin

The resistance of *Gardnerella* spp. to metronidazole and clindamycin before and after biofilm formation is shown in Table 3. Planktonic *Gardnerella* spp. isolates had a significantly higher susceptibility rate to clindamycin than metronidazole (87.5% vs. 25%, respectively) and a lower resistance rate (12.5% vs. 75%, respectively). These findings indicated that clindamycin is a better choice to eliminate *Gardnerella* spp. than metronidazole. *Gardnerella* biofilms had a higher MIC in response to metronidazole than planktonic isolates. Meanwhile, *Gardnerella* biofilms had a higher resistance to clindamycin than planktonic *Gardnerella*. The resistance of the cpn60 subgroups did not differ in response to metronidazole and clindamycin treatment for planktonic *Gardnerella* spp. (*p* >0.05).

### 3.6. Genomic Extraction of Gardnerella Isolates and Detection of Virulence Factor-Related Genes

The basic characteristics of the four *Gardnerella* isolate genomes are shown in Table 4. The genome ranged in size from 1.54 to 1.75 Mb. The GC content of the *Gardnerella* isolates was 41–43%, and each strain contained approximately 1300 genes. The sequences of plasmids, self-replicating DNA molecules that exist in prokaryotic cells independent of nuclear DNA, did not differ between the four strains. Non-coding RNAs (ncRNAs) are non-protein-coding RNAs, including tRNA, rRNA, snRNA, and siRNA, that are transcribed from the genome and function as direct RNA molecules. All four strains had the same amount of tRNA (n = 45). The CRISPR sequence was detected in S20 and S23 but not in S24 or S25 (Table 4). The KEGG functions of the four strains were primarily related to metabolism and genetic information processing (Table 4). There were significantly fewer genes involved in metabolism in S24 and S25 than in S20 and S23. The reduced involvement of genes involved in carbohydrate synthesis and metabolism may explain why S24 and S25 have less ability to form biofilms (Table 4). Meanwhile, S20 and S23, which have a strong ability to form biofilms, contain HMPREF0424_0103, HMPREF0424_1109, HMPREF0424_0125, and HMPREF0424_0821 (Table 4).

## 4. Discussion

Clue cells, vaginal squamous epithelial cells coated with *Gardnerella* spp. and other anaerobic bacteria, result from biofilms [30,31,32]. Interestingly, the current study found that women with clue cell-negative BV were more easily cured of the disease. While clue cells are used to confirm dysbiosis associated with BV, more than 60 years have passed since their first description and no consensus exists about how best to define them [33]. In addition, clue cell taxon indifferent imaging does not allow for exact analysis of the microbial layer adjacent to vaginal epithelial cells [34]. The simplistic view of BV as a dysbiosis, characterized by microscopic reference methods, has failed to inform the development of effective treatments [35].

BV is associated with a highly structured polymicrobial biofilm on the vaginal epithelium [36,37]. These biofilms protect BV-associated bacteria against antibiotics and promote disease recurrence [38]. The formation of *Gardnerella* spp. -dominated vaginal biofilms is a critical pathogenic agent in BV [39]. Biofilm formation is a continuous process of adhesion, coaggregation, maturation, and dispersion [38]. After biofilm maturation, it becomes difficult for the immune system to effectively clear the infection and standard antibiotics fail to completely eliminate the bacteria [40]. This explains why the rate of recurrence is >50% [41]. Consistent with these findings, our study found that natural dissipation of the biofilms did not occur after maturation in vitro. The biofilms were cultured in vivo for 48 h to reach a stable stage.

*Gardnerella* spp. is divided into four subgroups (A, B, C, and D) based on cpn60 barcode sequences [42,43] or classified into clades 1, 2, 3, and 4 by qPCR sequencing [44]. Subgroups A, B, C, and D correspond to clades 4, 2, 1, and 3, respectively [45]. Previous studies have confirmed that clades 4, 1, and 3 (Subgroups A, C, and D) are more often associated with BV [46,47]. Subgroup B, or clade 2, is more abundant in women with an intermediate Nugent score [45,46,47]. These studies highlight the potential clinical significance of *Gardnerella* spp. subgroups.

No differences were detected in the bacterial resistance of each subgroup; however, clindamycin was more effective than metronidazole in treating BV patients. *Gardnerella* clade 3 (subgroup D) and clade 4 (subgroup A) strains were previously shown to have 100% metronidazole resistance, while clade 1 (subgroup C) and clade 2 (subgroup B) had 35% and 7.1% metronidazole resistance, respectively [43]. The formation of biofilm is an important factor involved in *Gardnerella* spp. resistance [48,49]. BV women are often infected with multiple subtypes of *Gardnerella* spp. [50,51,52] and antibiotics typically eliminate sensitive *Gardnerella* subtypes but allow drug-resistant subtypes to survive, promoting treatment failure and disease recurrence [53,54].

RNA sequencing has shown that *Gardnerella* strains that form biofilms often have decreased metabolic activity, allowing for biofilm persistence. Cas genes are highly upregulated in treatment-resistant strains [55]. These genes also protect against phages and may be involved in DNA repair, mitigating the bactericidal effect of DNA-damaging agents, such as metronidazole [55]. The use of CRISPR-engineered phages for the treatment of dysbiosis may provide a deeper understanding of the human microbiome and inform the development of novel treatment options [56]. Environmental pressures or ecological disturbances of the vaginal niche may influence biofilm formation and the development of BV more than the *Gardnerella* spp. genotype alone [57]. However, it is important to note that the expression of biofilm-forming genes can promote biofilm formation in vitro.

In conclusion, multiple factors may contribute to the failure of current BV treatments. Subsequent studies using a larger sample size are needed to explore the relationship between BV treatment and strain pathogenicity.

## 5. Limitations

This study had several limitations. First, the number of isolated strains was insufficient. Three samples with unqualified DNA samples were excluded, allowing only eight strains to be sequenced for cpn60. Second, microtiter plate assays do not accurately represent in vivo conditions and growth media does not contain all factors associated with an in vivo infection [58,59]. Finally, this study only focused on the biofilm of a single *Gardnerella* species. Biofilms in the female vagina are usually composed of many species that interact synergistically to evade treatment, helping to explain the clinically high rates of BV recurrence [39].

## Figures and Tables

**Table 1:** Clinical and phenotypic characteristics of the *Gardnerella* strains used for cpn60 sequencing.

Clinical Isolate	Age	Symptoms	Treatment BT/AT	Clue Cells BT/AT	Nugent * Scores	Diagnosis	Treatment Outcomes
S2	44	Abnormal discharge	BT	+/+	8	BV	Uncured
S5	39	Abnormal discharge, odor	BT	−/−	10	BV	Cure
S9	26	Abnormal discharge	BT	−/−	8	BV	Cure
S10	42	Abnormal discharge	BT	−/−	8	BV	Cure
S13	38	Abnormal discharge, itching	BT	+/−	7	BV	Cure
S18	31	Abnormal discharge	BT	+/+	7	BV	Uncured
S25	37	Abnormal discharge	BT	+/+	8	BV	Uncured
S27	49	Abnormal discharge	BT	+/−	8	BV	Uncured
S20	41	None	AT	+/−	4	Intermediate	−
S23	36	Abnormal discharge	AT	−/−	1	Nomal	−
S24	24	None	AT	−/−	0	Nomal	−

* A Nugent score of 0–3 is consistent with a *Lactobacillus*-predominant vaginal microbiota, a score of 4–6 correlates with intermediate microbiota (the emergence of *Gardnerella* spp.), and a score of 7–10 correlates with BV. After treatment, a patient with a Nugent score of 0–3 is considered cured and a patient with a Nugent score of 4–10 is considered uncured. +, cue cell positive; −, cue cell negative. BT, before treatment; AT after treatment. −, without result. Samples from after treatment and known treatment outcome.

**Table 2:** *Gardnerella* spp. subgroups created by cpn60 sequencing and biofilm formation ability.

cpn60 Typing	Clincal Isolate	Biofilm (Mean ± SD) (OD_595_)	Mean in Subgroup (Mean ± SD) (OD_595_)
Subgroup A	S2	1.262 ± 0.152 (S)	0.7223 ± 0.4293
	S9	0.627 ± 0.029 (M)	
	S10	1.244 ± 0.026 (S)	
	S13	0.449 ± 0.058 (W)	
	S18	0.507 ± 0.065 (M)	
	S25	0.245 ± 0.023 (W)	
Subgroup B	S27	1.567 ± 0.019 (S)	−
Subgroup D	S5	2.132 ± 0.072 (S)	−

N, no biofilm producer; W, weak biofilm producer; M, moderate biofilm producer; S, strong biofilm producer. Mann–Whitney test: Subgroup A vs. B, *p* = 0.003; Subgroup A vs. D, *p* < 0.001; Subgroup B vs. D, *p* < 0.001. −, without result. The mean could not be calculated.

**Table 3:** Susceptibility of *Gardnerella* strains to metronidazole and clindamycin before and after biofilm formation.

Subgroup	Strains	Biofilm	Metronidazole (μg/mL) MIC			Clindamycin (μg/mL) MIC		
			Planktonic Cells	24 h Biofilm	48 h Biofilm	Planktonic Cells	24 h Biofilm	48 h Biofilm
Subgroup A	S2	S	<0.125	1	1	<0.0625	<0.0625	0.125
	S9	M	128	>256	>256	<0.0625	>128	>128
	S10	S	64	128	256	0.25	>128	>128
	S13	W	128	>256	>256	<0.0625	>128	>128
	S18	M	128	32	>256	64	>128	>128
	S25	W	<0.125	1	1	<0.0625	<0.0625	0.125
Subgroup B	S27	S	128	256	256	<0.0625	0.125	0.125
Subgroup D	S5	S	64	64	128	<0.0625	<0.0625	0.125

Using CLSI guidelines, metronidazole and clindamycin have the following critical values: metronidazole (sensitivity: MIC ≤ 8 μg/mL; intermediate: MIC = 16 μg/mL; drug-resistant: MIC ≥ 32 μg/mL) and clindamycin (sensitivity: MIC ≤ 2 μg/mL; intermediate: MIC = 4 μg/mL; resistant: MIC ≥ 8 μg/mL). *T*-test: Biofilm formation did not differ significantly between the planktonic state of subgroups A vs. B/D after treatment with metronidazole (*p* = 0.576); subgroups B vs. D were not statistically different. Biofilm formation after 24 h and 48 h was similar for subgroup A vs. B/D (*p* = 0.076 and *p* = 0.175, respectively). Biofilm formation did not differ significantly between the planktonic state of subgroups A vs. B/D after treatment with clindamycin (*p* = 0.363); subgroups B vs. D were not statistically significant. Biofilm formation after 24 h and 48 h differed for subgroups A vs. B/D (*p* = 0.025 for both). The MIC of *Gardnerella* spp. in the planktonic state was similar for metronidazole and clindamycin (*p* = 0.286). After 24 h and 48 h, the MIC of *Gardnerella* spp. biofilms were similar in response to metronidazole and clindamycin (*p* = 0.437 and *p* = 0.576, respectively).

**Table 4:** Characteristics of the four *Gardnerella* strains.

Strains	S20	S23	S24	S25
Size(bp)	1,686,096	1,685,505	1,549,419	1,582,566
CDS	1316	1359	1290	1286
Plasmid	NO	NO	NO	NO
Island	6	6	6	6
GC %	41.31	41.81	42.55	42.74
tRNA	45	45	45	45
rRNA	6	6	6	6
CRISPER	1	1	0	0
Metabolism	376	414	281	259
Genetic Information Processing	276	214	270	208
Environmental Information Processing	82	80	114	106
Cellular Processes	58	58	102	110
Organismal Systems	28	19	36	25
Human Diseases	31	30	62	44
HMPREF0424_0103	+	+	−	−
HMPREF0424_1109	+	+	−	−
HMPREF0424_0125	+	+	−	−
HMPREF0424_0821	+	+	−	−

+, gene positive; −, gene negative.

## Data Availability

All data presented in this study are available on request by contacting the corresponding author.

## Supplementary Materials

The following supporting information can be downloaded at: https://www.mdpi.com/article/10.3390/microorganisms11092186/s1, Table S1: Primer of virulence factor related genes in biofilm formation.
