# Supplementary material for: Biofilm-Forming Capacity and Drug Resistance of Different Gardnerella Subgroups Associated with Bacterial Vaginosis
Source: Microorganisms. 2023 Aug 30;11(9):2186. doi: 10.3390/microorganisms11092186 (PMC10534620; doi:10.3390/microorganisms11092186)
Supplement: Supplementary file 1 [file microorganisms-11-02186-s001.zip › microorganisms-2416378-supplementary.docx]

**Supplementary Table 1.** Primers of virulence factor-related genes involved in biofilm formation

| **Genes** | **Product** | **Sequences** | **Function** |
| --- | --- | --- | --- |
| HMPREF0424_1109 F | Sialidase F | CCGAATTTGCGATTTCTTCT | Mucin degradation |
| HMPREF0424_1109 R | Sialidase R | CGTACGGAAGTTTTGGAAGC |  |
| HMPREF0424_0103 F | Vaginolysin F, (vly) | CTCGCATGCAGTACGATTCT | Cytotoxicity |
| HMPREF0424_0103 R | Vaginolysin R, (vly) | TCTGGTGCATCAACGCTTAC |  |
| HMPREF0424_0125 F | TadE-like protein F, (Tdr) F | GCAAGAATACTCTGGACGAGGT | Epithelial adhesion |
| HMPREF0424_0125 R | TadE-like protein R, (Tdr) R | CAAAAGCTGAATCGCCTCCG |  |
| HMPREF0424_0821 F | Family 2 glycosyltransferase F | CAACGAAGGCATAGGTTTCC | EPS production and Biofilm formation |
| HMPREF0424_0821 R | Family 2 glycosyltransferase R | GCGCTTGGAACTGCTTTAAC |  |
